# Association of Adiponectin with High-Sensitivity C-Reactive Protein and Clinical Outcomes in Peritoneal Dialysis Patients: A 3.5-Year Follow-Up Study

**DOI:** 10.1371/journal.pone.0141058

**Published:** 2015-10-16

**Authors:** Chun-Wu Tung, Yung-Chien Hsu, Ya-Hsueh Shih, Chun-Liang Lin

**Affiliations:** 1 Department of Nephrology, Chang Gung Memorial Hospital, Chiayi, Taiwan; 2 Graduate Institute of Clinical Medical Science, College of Medicine, Chang Gung University, Taoyuan, Taiwan; 3 Department of Kidney and Diabetic Complications Research Team (KDCRT), Chang Gung Memorial Hospital, Chiayi, Taiwan; 4 Chronic Kidney Disease Center, Chang Gung Memorial Hospital, Chiayi, Taiwan; 5 School of Traditional Chinese Medicine, College of Medicine, Chang Gung University, Taoyuan, Taiwan, Republic of China; Morehouse School of Medicine, UNITED STATES

## Abstract

**Introduction:**

Adiponectin (ADPN), one of most abundant fat-derived biologically active substances, plays an important role in anti-atherosclerotic process. There are conflicting results about the impact of ADPN on cardiovascular (CV) outcomes and mortality, particularly in patients undergoing peritoneal dialysis (PD). Moreover, the relationship between ADPN and inflammatory mediators has been seldom explored in this population. Therefore, we examined the relationship between ADPN and longitudinal high-sensitivity C-reactive protein (hs-CRP) changes and investigated whether ADPN or hs-CRP levels could predict CV outcomes and mortality in prevalent PD patients after comprehensive adjustment of possible confounders.

**Methods:**

In this prospective cohort study, 78 PD patients were enrolled and followed from February 2009 to August 2012. During follow-up, CV events and all-cause mortality were recorded.

**Results:**

The mean baseline ADPN value was 29.46±18.01 μg/ml and duration of PD treatment was 37.76±36.96 months. In multiple linear regression analysis, plasma ADPN levels positively correlated with high-density lipoprotein and negatively associated with hs-CRP, body mass index, D4/D0 glucose, triglyceride, and duration of PD treatment. After stratified by genders, the inverse association between baseline ADPN and hs-CRP was more significant in the female group. The hs-CRP levels were followed up annually and remained significantly lower in the high ADPN group in the first 2 years. Patients were then stratified into two groups according to the median ADPN value (23.8 μg/ml). The results of Kaplan-Meier survival analysis demonstrated less CV events and better survival in high ADPN group. On multivariate Cox regression analysis, only ADPN level (HR: 0.93, 95% CI: 0.88–0.98, p = 0.02), age and history of CV diseases were independent risk factors for future CV events. Furthermore, hs-CRP (HR: 1.11, 95% CI:1.001–1.22, p = 0.04) was identified as independent predictor of all-cause mortality.

**Conclusions:**

Serum hs-CRP levels were consistently lower in the high ADPN group during 2-year follow-up. We also demonstrated the importance of ADPN and hs-CRP in predicting CV events and all-cause mortality in PD population during 3.5-year follow-up.

## Introduction

Peritoneal dialysis (PD) is a well-established renal replacement therapeutic modality for patients with end-stage renal disease (ESRD). Patients on PD treatment may have better quality of life, independence [1] and lower total healthcare costs than hemodialysis (HD) [2,3]. However, the long term survival rate of PD patient is still poor with median survival less than 5 years [4] despite enormous reduction of PD peritonitis rate in the past decades. Cardiovascular (CV) diseases are the most common causes of death in dialysis patients. After one year of treatment, PD therapy was consistently associated with greater CV mortality compared with HD [5,6]. Undesirable inflammatory and metabolic consequences will develop after long-term exposure to bio-incompatible dialysis solutions [7]. Previous studies had showed uremia and PD therapy bring them more pronounced metabolic abnormalities in the atherogenic process and thus higher CV mortality than HD [8].

Adiponectin (ADPN), one of most abundant fat-derived biologically active substances, has recently attracted much attention because of its insulin-sensitizing [9,10] and anti-atherosclerotic properties [11]. Levels of ADPN, in contrast to other adipocytokines, are reduced in obese patients and those with diabetes mellitus. Consequently, high ADPN concentrations are associated with a favorable CV outcomes in general populations [12]. However, high levels of plasma ADPN have also been shown to be a predictor of increased all-cause and CV mortalities in some patients with established CV diseases [13–15]. It would become more complex when analyzing the role of ADPN on CV outcomes and mortality in patients with renal impairment. Because abnormalities of glucose or lipoprotein metabolism and insulin resistance are common in patients with chronic kidney disease, gradual reduction of ADPN along with progression of renal failure would be expected. In contrast, ADPN is inversely correlated to glomerular filtration rate partly due to reduced renal clearance. Plasma ADPN has been shown to be increased in ESRD patients on PD or HD therapies [16,17]. Despite intensive studies in the past, the impact of ADPN on the CV outcome and survival in dialysis patients remains unclear. Zoccali et al. had suggested higher ADPN concentrations are inversely related to incident CV events [16,18] and mortality risk in HD patients. In contrary, recent data indicated that elevation of ADPN was associated with adverse CV outcomes and increased mortality in patients with chronic kidney disease [19] and those on HD treatment [20,21]. There are also contradictory results about the relationship between circulating ADPN levels and clinical outcomes in patients undergoing PD therapy [22,23]. These discordant findings may be caused by the differences in study designs, ethnic background and residual confounders relating to inconsistent covariate adjustment. Adiponectin might also act as an effective anti-inflammatory protein for reducing endothelial damage [24,25], and chronic inflammation in uremic patients is significantly associated with atherosclerosis. However, the relationship between ADPN and inflammatory mediators has been seldom explored in PD patients. C-reactive protein (CRP), an acute phase reactant, has been shown to be an independent predictor of CV events and mortality in PD patients [26,27]. We used a high-sensitivity CRP (hs-CRP) test, which is more sensitive than conventional CRP examination, to clarify the associations between ADPN and chronic inflammation.

The aim of this prospective cohort study was to examine the relationship between ADPN and longitudinal hs-CRP changes and to investigate whether circulating ADPN and hs-CRP levels were independent predictors for CV events and mortality in prevalent PD patients.

## Materials and Methods

### Study Population

This prospective cohort study adhered to the guidelines outlined in the Declaration of Helsinki. All participants at study entry provided written informed consent, and the Medical Ethics Committee of Chang Gung Memorial Hospital approved this study (Institutional Review Board number: 99-0511B). This study was conducted in the PD center of Chang Gung Memorial Hospital, Chiayi, Taiwan from February 2009 to August 2012.

Uremic patients undergoing continuous ambulatory PD with the twin-bag system (Ultrabag; Baxter Healthcare, Singapore) or continuous cyclic PD with a cycler (Home-Choice; Baxter Healthcare, McGaw Park, IL, USA) for more than 6 months were enrolled in this study. Among 90 patients, 12 patients were excluded owing to the following reasons: (1) unstable medical conditions or active infections such as acute myocardial infarction, acute stroke, pneumonia or peritonitis within 3 months; (2) decompensated liver diseases; (3) concurrent malignancy; (4) patients’ refusal or incomplete data records.

### Baseline Demographic and Clinical Data

Baseline data, including age, gender, past medical histories (diabetes mellitus, hypertension, ischemic heart disease and ischemic stroke), duration of PD therapy at the entry of this study, and body mass index (BMI) of each patient were recorded. Concurrent medications (anti-hypertensive drugs, statins, anti-platelet agents and calcitriol) and the dose of monthly erythropoietin were also documented. All patients were asked to continue their regular medications during this study period. Systolic and diastolic blood pressures were computed as the mean of three most recent monthly measurements. Body weight and blood pressure were measured before dialysate instillation.

### Measurements of Laboratory Parameters

After a minimum 8-hour overnight fast, venous blood were sampled before the first daily dwell of dialysate. It was drawn into EDTA tubes, centrifuged at 4°C immediately, and then stored at -80°C in aliquots until further assay analyses. Serum intact parathyroid hormone (i-PTH) was determined by utilizing a chemiluminometric immunoassay (ADVIA Centaur i-PTH; Siemens Medical Solutions Diagnostics, New York, NY, USA). Plasma ADPN level was analyzed by using an enzyme-linked immunosorbent assay kit (R &D System, Minneapolis, MN, USA). The ADPN concentrations were determined by the average of two separate sample measurements. We also measured hs-CRP by immunonephelometric method (Nanopia CRP; Daiichi, Tokyo, Japan) at the entry of this study and annually.

Hemogram and other biochemical parameter, including blood sugar, blood urea nitrogen (BUN), serum creatinine (Cr), calcium, phosphorus, uric acid, albumin, ferritin, glycated hemoglobin, triglyceride (TG), total cholesterol, low-density lipoprotein (LDL-C), and high-density lipoprotein (HDL-C) were obtained through an automatic analyzer following standard laboratory procedures at the Department of Laboratory Medicine at Chiayi Chang Gung Memorial Hospital.

### Peritoneal Dialysis Membrane Characteristics

The transport characteristics of the peritoneal membrane were accessed with standard peritoneal equilibration test (PET) as described by Twardowski [28]. All patients were in euvolemic state during PET and it was performed with 2 liters of 2.5% dextrose solution. Dialysate samples, D0 and D4, were obtained promptly after completion of instillation and at 4 hours after drainage, respectively. Dialysate to plasma ratio at 4 hours for Cr (D/P Cr) was used to quantify individual peritoneal membrane characteristics. Additionally, 24-hour dialysate effluent and urine samples were collected for measurement of residual renal function, solute clearance and normalized protein nitrogen appearance (nPNA). Weekly renal, peritoneal and total Kt/V urea and nPNA were calculated by a computer program (PD adequest; Baxter Healthcare, McGaw Park, IL, USA). Residual renal glomerular filtration rate was estimated by the average of renal creatinine and urea nitrogen clearance rates.

### Outcome Measures

Cardiovascular events were defined as fatal or non-fatal myocardial infarction, intervention of coronary artery disease with angioplasty or stenting, acute ischemic stroke and peripheral vascular disease requiring arterial revascularization. Aforementioned outcomes were ascertained from medical chart, and angiographic or brain image studies.

No patient was lost to follow up during the 3.5-year observational period. Patients who underwent kidney transplantation or were transferred to hemodialysis were censored in survival or CV events analysis.

### Statistical Analysis

Descriptive results of continuous variables are expressed as means ± standard deviations (SD) while categorical data are reported as percentages and numbers. Patients were divided into two groups according to their baseline median ADPN value. Differences between the two groups were analyzed via Mann–Whitney *U*-test for quantitative variables and chi-square with Fisher’s exact test for categorical variables. Values of ADPN and hs-CRP were transformed to natural logarithms before association analysis. The relations between ADPN and other independent variables were analyzed by simple and stepwise backward multivariate linear regression analyses, adjusting significant factors with a p value less than 0.10. After 3.5-year follow-up period, cumulative CV events and mortality between the two groups were examined by Kaplan–Meier survival analyses with long-rank test. Univariate Cox proportional hazards regression was applied to estimate the relative risk of possible factors associated with mortality and CV events. To adjust confounding factors, multivariate Cox regression analysis with stepwise backward approach was performed (factors with p value < 0.10 on the univariate analysis were added in). These statistical analyses were performed using SPSS 15.0 statistical software for Windows (SPSS Inc, Chicago, IL, USA). Any p values less than 0.05 were considered statistically significant.

## Results

### Baseline Characteristics of Demography and Biochemistry

This study cohort consisted of 78 patients with a mean age of 52.14±12.93 years, of whom 50% were male. The mean ADPN value was 29.46±18.01 μg/ml and duration of PD treatment was 37.76±36.96 months. Diabetes, hypertension and CVD were detected in 24 (30.8%), 58 (74.4%) and 12 (15.4%) patients, respectively. Most of them took anti-hypertensive agents (calcium channel blockers, n = 41; angiotensin receptor blockade/angiotensin converting enzyme inhibitors, n = 32; and β-blockers, n = 38), statins (n = 34), and calcitriol (n = 31). Eighteen patients (23.1%) were on anti-platelet drugs, including aspirin or clopidogrel. Other baseline characteristics, including properties of peritoneal membrane, adequacy of PD therapy, and biochemical data of study population are shown in Table 1.

**Table 1:** Characteristics of study population and correlation between plasma adiponectin levels and clinical variables at baseline.

	All (N = 78)	Pearson r	p value for r
Adiponectin, μg/mL	29.46±18.01		
**Demographics**			
Age, years	52.14±12.93	-0.403	<0.01
Gender, (female/male)	39/39		
Duration of PD, months	37.76±36.96	-0.215	0.04
Body mass index, kg/m^2^	23.00±3.36	-0.401	<0.01
Systolic blood pressure, mmHg	134.69±18.25	0.169	0.14
Diastolic blood pressure, mmHg	80.24±10.21	0.371	0.04
**Concomitant diseases, % (n)**			
Diabetes mellitus	30.8 (24)		
Hypertension	74.4 (58)		
Ischemic heart disease/ stroke	15.4 (12)		
**Dialysis Parameters**			
*Adequacy of dialysis*			
Solute clearance (Total Kt/V)	2.12±0.31	-0.149	0.20
Peritoneal Kt/V	1.86±0.37	-0.049	0.67
Residual GFR (ml/min per 1.73 m^2^)	1.72±2.40	-0.076	0.51
Renal Kt/V	0.26±0.39	-0.072	0.54
nPNA (gm/Kg/day)	1.09±0.24	0.180	0.12
Daily Urine Amount, mL	347.37±382.72	0.222	0.06
*Peritoneal solute transport rate*			
D4/D0 Glucose	0.39±0.06	-0.220	0.05
4-h D/P Cr	0.67±0.09	0.177	0.13
**Biochemical parameters**			
White blood cell count, 10^3^ cells/uL	8.24±4.06	-0.044	0.70
Hemoglobin, g/dL	10.04±1.47	0.020	0.87
Albumin, g/dL	3.82±0.44	-0.138	0.23
Total cholesterol, mg/dL	184.37±41.54	0.051	0.66
LDL-C, mg/dL	105.29±37.15	0.121	0.29
HDL-C, mg/dL	43.43±16.54	0.366	<0.01
Triglyceride, mg/dL	189.40±130.86	-0.348	<0.01
Hemoglobin A1c, %	5.99±1.23	-0.046	0.69
Fasting glucose, mg/dL	125.17±49.64	-0.041	0.72
BUN, mg/dL	62.14±17.30	0.127	0.27
Creatinine, mg/dL	12.53±3.25	-0.051	0.66
Uric acid, mg/dL	7.11±1.42	-0.268	0.02
Calcium, mg/dL	9.41±0.82	-0.238	0.04
Phosphorus, mg/dL	4.82±0.50	0.017	0.88
Ferritin, μg/L	572.59±459.21	-0.216	0.06
i-PTH, pg/mL	274.89±255.17	-0.070	0.55
**Medications, % (n)**			
Calcium channel blocker	52.6 (41)		
ARB/ACEI	41 (32)		
Beta-blocker	48.7 (38)		
Statins	43.6 (34)		
Anti-platelet agents	23.1 (18)		
Calcitriol	39.7 (31)		
EPO, Units/month	13378.38±7453.42	-0.108	0.38
Hs-CRP, mg/L	4.08±4.41	-0.412	<0.01

Values expressed as mean ± standard deviation or percentage (number)

Abbreviations: PD, peritoneal dialysis; GFR, glomerular filtration rate; nPNA, normalized protein nitrogen appearance; 4-h D/P Cr, dialysate/plasma creatinine ratio at 4 hours; LDL-C, low-density lipoprotein; HDL-C, high-density lipoprotein; BUN, blood urea nitrogen; i-PTH, intact-parathyroid hormone; ARB/ACEI, angiotensin receptor blockade/angiotensin converting enzyme inhibitors; EPO, erythropoietin; hs-CRP, high sensitivity C-reactive protein at baseline.

### Determinants of Plasma Adiponectin Levels

The correlation between serum ADPN and continuous parameters were analyzed by Pearson's correlation method. Serum ADPN was positively related to diastolic blood pressure and HDL-C and inversely associated with age, duration of PD treatment, BMI, D4/D0 glucose ratio, TG, uric acid, calcium and hs-CRP levels (Table 1). In multiple linear regression analysis, where variables significant in univariate analysis were included, lower hs-CRP (β = −0.085 ± 0.049, p = 0.045), BMI (β = −0.057 ± 0.017, p = 0.001), D4/D0 glucose ratio (β = −3.683 ± 1.047, p = 0.001), TG (β = −0.001 ± 0.000, p = 0.016) and shorter duration of PD treatment (β = −0.005 ± 0.002, p = 0.004) and higher HDL-C (β = 0.019 ± 0.005, p = 0.001) were notably associated with higher ADPN levels (R^2^ = 0.512, Table 2). In order to determine whether gender status influenced the relations between adiponectin, hs-CRP and other clinical variables, further simple and multivariate linear regression analyses were conducted for both males and females. S1 Table summarizes the clinical and biochemical characteristics of the male and female subgroups. The females had higher total Kt/V, nPNA, total cholesterol, HDL-cholesterol and lower serum creatinine levels than males. There was comparable ADPN and hs-CRP values between groups. As shown in S2 Table, the baseline hs-CRP was negatively associated with ADPN in both genders (Male: r = -0.342, p = 0.02; Female: r = -0.477, p<0.01). However, the results of multiple linear regression revealed that the negative correlation between hs-CRP and ADPN was more significant in women (Male: β = −0.116 ± 0.066, p = 0.086; Female: β = −0.142 ± 0.078, p = 0.01, Table 2).

**Table 2:** Relationship between adiponectin, hs-CRP and baseline characteristics according to gender by multiple linear regression.

	Ln Adiponectin								
	Male			Female			Overall		
	β	SE	p	β	SE	p	β	SE	p
Constant	2.828	0.843	0.002	2.930	0.733	<0.001	4.896	0.738	<0.001
Ln hs-CRP	-0.116	0.066	0.086	-0.142	0.078	0.01	-0.085	0.049	0.045
Duration of PD (mo)				-0.003	0.002	0.04	-0.005	0.002	0.04
BMI (Kg/m^2^)				-0.051	0.024	0.002	-0.057	0.017	0.001
D4/D0 Glu	-3.988	1.526	0.014				-3.683	1.047	0.001
HDL-C (mg/dL)				0.013	0.006	0.001	0.019	0.005	0.001
TG (mg/dL)				-0.003	0.001	0.002	-0.001	0.000	0.016
Calcium (mg/dL)	-0.252	0.104	0.021						
DBP (mmHg)				0.019	0.009	0.04			
Daily urine (mL)	0.001	0.001	0.003						
R^2^	0.470			0.589			0.512		

hs-CRP, high sensitivity C-reactive protein at baseline; PD, peritoneal dialysis; BMI, body mass index; HDL-C, high-density lipoprotein; TG: triglyceride; DBP, diastolic blood pressure.

### Characteristics between Low versus High Adiponectin Population

We divided patients into high and low ADPN level groups according to the median ADPN value (23.8 μg/ml). The patients’ characteristics between low versus high ADPN levels were summarized in Table 3. The mean ADPN concentrations of low and high ADPN group are equal to 15.83±5.20 and 43.09±15.79 μg/ml respectively. The two groups did not differ significantly from each other in terms of age, sex, PD duration, systolic blood pressure, prevalence of diabetes mellitus, hypertension and CV disease, adequacy of dialysis therapy, residual renal function, protein intake (nPNA), peritoneal membrane characteristics, white blood cell counts, hemoglobin, Albumin, BUN, serum creatinine, uric acid, glycemic control, calcium, phosphorus and i-PTH. There was also no statistical difference about the use of anti-hypertensive drugs, statins, anti-platelet agents, calcitriol and erythropoietin doses between groups.

**Table 3:** Patient characteristics according to different adiponectin levels.

	Low-adiponectin group <23.8 μg/mL (N = 39)	High-adiponectin group ≧23.8 μg/mL (N = 39)	p value
Adiponectin, μg/mL	15.83±5.20	43.09±15.79	< 0.01
**Demographics**			
Age, years	54.28±11.78	50.00±13.80	0.15
Gender, (female/male)	17/22 (43.6%)	22/17 (56.4%)	0.26
Duration of PD, months	43.77±43.47	31.74±28.36	0.15
Body mass index, kg/m^2^	24.24±3.13	21.73±3.13	< 0.01
Systolic blood pressure, mmHg	132.38±19.14	137.00±17.25	0.27
Diastolic blood pressure, mmHg	77.69±10.43	82.79±9.43	0.03
**Concomitant diseases, % (n)**			
Diabetes mellitus	30.8 (12)	30.8 (12)	1.00
Hypertension	71.8 (28)	76.9 (30)	0.60
Ischemic heart disease/stroke	17.9 (7)	12.8 (5)	0.76
**Dialysis Parameters**			
*Adequacy of dialysis*			
Solute clearance (Total Kt/V)	2.18±0.31	2.06±0.30	0.11
Peritoneal Kt/V	1.89±0.41	1.83±0.33	0.46
Residual GFR (ml/min per 1.73 m^2^)	1.83±3.08	1.61±1.47	0.12
Renal Kt/V	0.29±0.51	0.23±0.21	0.12
nPNA (gm/Kg/day)	1.08±0.26	1.11±0.22	0.61
Daily Urine Amount, mL	299.47±426.05	395.26±332.69	0.04
*Peritoneal solute transport rate*			
D4/D0 Glu	0.40±0.06	0.39±0.06	0.48
4-h D/P Cr	0.67±0.09	0.67±0.09	0.84
**Biochemical parameters**			
White blood cell count, 10^3^ cells/uL	8.57±4.83	7.91±3.14	0.48
Hemoglobin, g/dL	10.01±1.39	10.07±1.57	0.84
Albumin, g/dL	3.87±0.49	3.77±0.38	0.29
Total cholesterol, mg/dL	180.41±46.13	188.33±36.56	0.4
LDL-C, mg/dL	100.18±42.49	110.53±30.41	0.22
HDL-C, mg/dL	38.97±14.23	48.00±17.65	0.02
Triglyceride, mg/dL	224.05±156.10	154.74±88.70	0.02
Hemoglobin A1c, %	6.06±1.23	5.92±1.26	0.62
Fasting glucose, mg/dL	127.59±48.21	122.74±51.55	0.67
BUN, mg/dL	60.69±14.25	63.59±19.97	0.46
Creatinine, mg/dL	12.52±3.36	12.53±3.18	0.99
Uric acid, mg/dL	7.37±1.32	6.84±1.49	0.07
Calcium, mg/dL	9.54±0.81	9.28±0.82	0.11
Phosphorus, mg/dL	4.86±1.01	4.79±1.04	0.75
Ferritin, μg/L	674.36±515.95	470.83±373.95	0.03
i-PTH, pg/mL	292.43±278.61	257.34±231.69	0.55
**Medications, % (n)**			
Calcium channel blocker	48.7 (19)	56.4 (22)	0.49
ARB/ACEI	38.5 (15)	43.6 (17)	0.65
Beta-blocker	59 (23)	41 (16)	0.11
Statins	48.7 (19)	38.5 (15)	0.36
Anti-platelet agents	25.6 (10)	20.5 (8)	0.79
Calcitriol	38.5 (15)	41 (16)	0.82
EPO, Units/month	14324.32±7280.32	12432.43±7603.15	0.28

Values expressed as mean ± standard deviation or percentage (number)

Abbreviations: PD, peritoneal dialysis; GFR, glomerular filtration rate; nPNA, normalized protein nitrogen appearance; 4-h D/P Cr, dialysate/plasma creatinine ratio at 4 hours; LDL-C, low-density lipoprotein; HDL-C, high-density lipoprotein; BUN, blood urea nitrogen; i-PTH, intact-parathyroid hormone; ARB/ACEI, angiotensin receptor blockade/angiotensin converting enzyme inhibitors; EPO, erythropoietin.

The examination of lipid profiles revealed a significant increase of HDL-C and reduction of TG levels in the high ADPN group, while no differences were noticed in total cholesterol and LDL-C. Additionally, higher adiponectin levels were associated lower BMI and ferritin levels, higher diastolic blood pressure and more daily urine amount.

### Association of Adiponectin and High-Sensitivity C Reactive Protein

**Fig 1:**
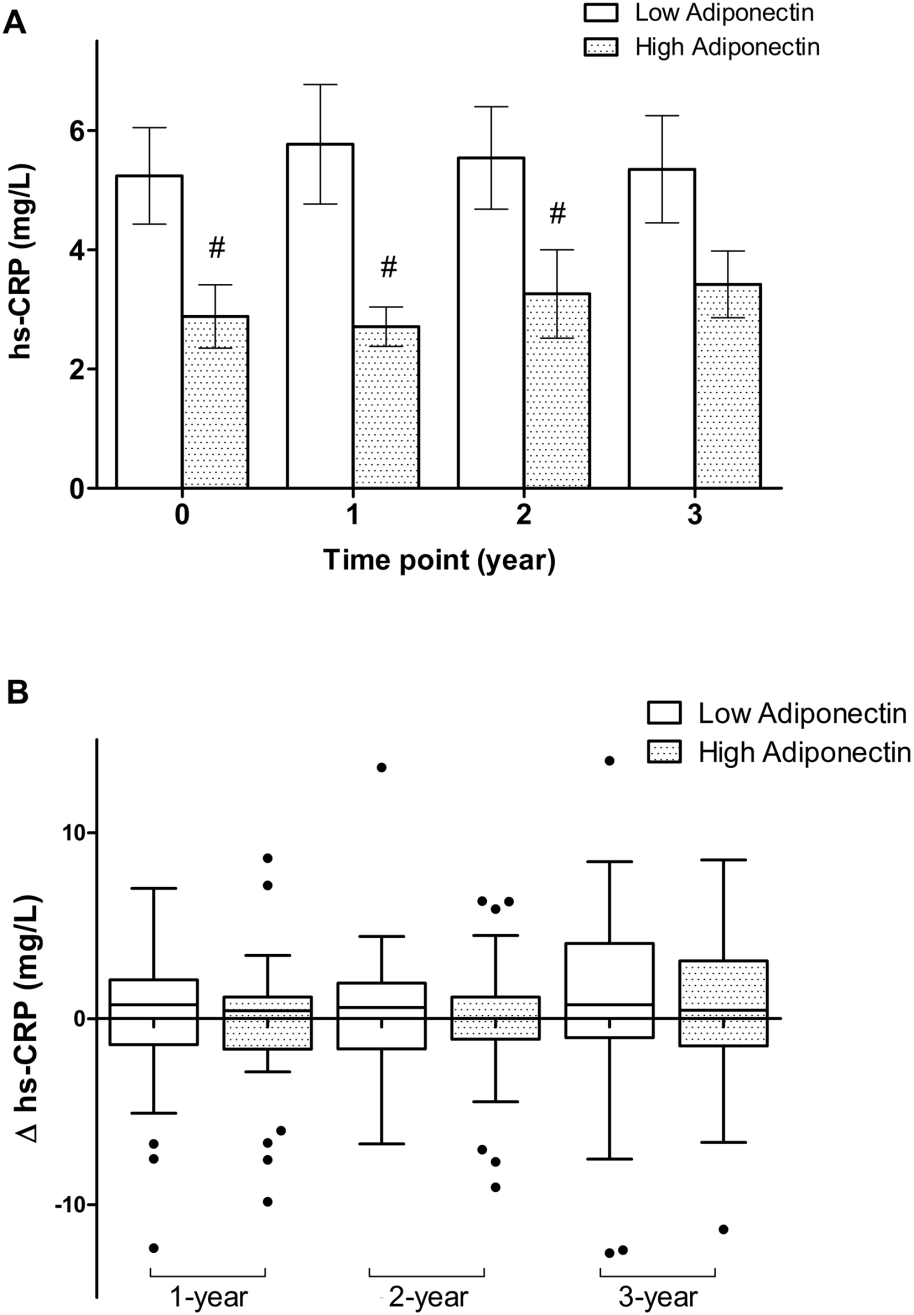
(A) Comparisons of high-sensitivity C-reactive protein (hs-CRP) levels between high and low adiponectin (ADPN) groups at indicated time point; (B) Box plots of changes of hs-CRP (Δhs-CRP) between high and low ADPN groups at indicated time point. # represent statically significant (p< 0.05) when compared to low ADPN group respectively.

**Fig 2:**
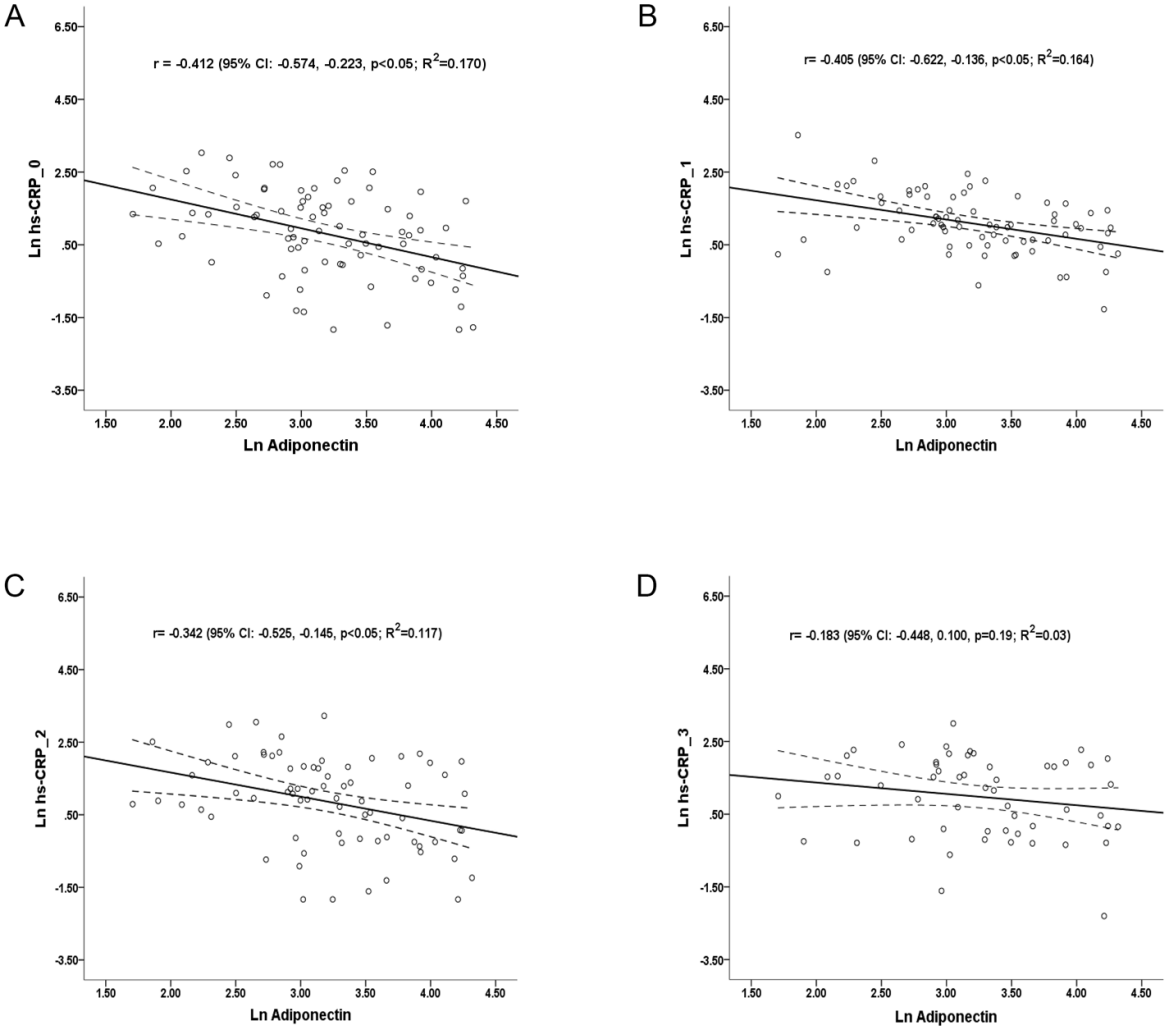
Scatter plots for correlations between baseline plasma ADPN and hs-CRP at baseline (A), one-year (B), 2 years (C) and 3 years later (D). The solid line is regression line, whereas dotted line represents the 95% confidence interval for the mean.

High-sensitivity CRP was checked at enrollment and followed annually. The hs-CRP level was significantly lower in the high ADPN group at baseline (2.88±3.24 vs. 5.24±5.08, p = 0.01), one-year follow up (2.71±2.02 vs. 5.77±5.91, p<0.01) and two years later (3.26±4.50 vs. 5.53±5.08, p = 0.01) (Fig 1A). Though there was no significant difference, the third-year hs-CRP tended to decrease among the high ADPN group (3.41±3.01 vs. 5.35±4.52, p = 0.12). The changes of hs-CRP between high and low ADPN groups were insignificant through the 3-year follow-up period (at one-year: -0.17±3.43 vs. 0.69±5.87, p = 0.43; at 2-year: 0.40±5.18 vs. 0.46±5.60, p = 0.39; and at 3-year: 0.42±4.18 vs. 0.94±5.83, p = 0.62, respectively) (Fig 1B) There was strong association between ADPN and hs-CRP initially (r = −0.412 (95% confidence interval (CI): −0.574, -0.223, p<0.01) (Fig 2A). Correlations remained statistically significant for the baseline ADPN and hs-CRP at one-year (r = −0.405, 95% CI: −0.622, -0.136, p< 0.01) and 2-year follow-up (r = −0.342, 95% CI: –0.525, -0.145, p< 0.01) (Fig 2B). The relationship between baseline ADPN and hs-CRP at 3-year later was less significant (r = −0.183, 95% CI: -0.448, 0.100, p = 0.19). There were similar expression values of hs-CRP among both genders during the 3-year follow-up period (S1 Table). Additionally, gender difference did not influence the inverse correlations between baseline ADPN and annually followed hs-CRP values (S2 Table).

### Baseline Adiponectin Concentration is an Independent Predictor of Cardiovascular Events

**Fig 3:**
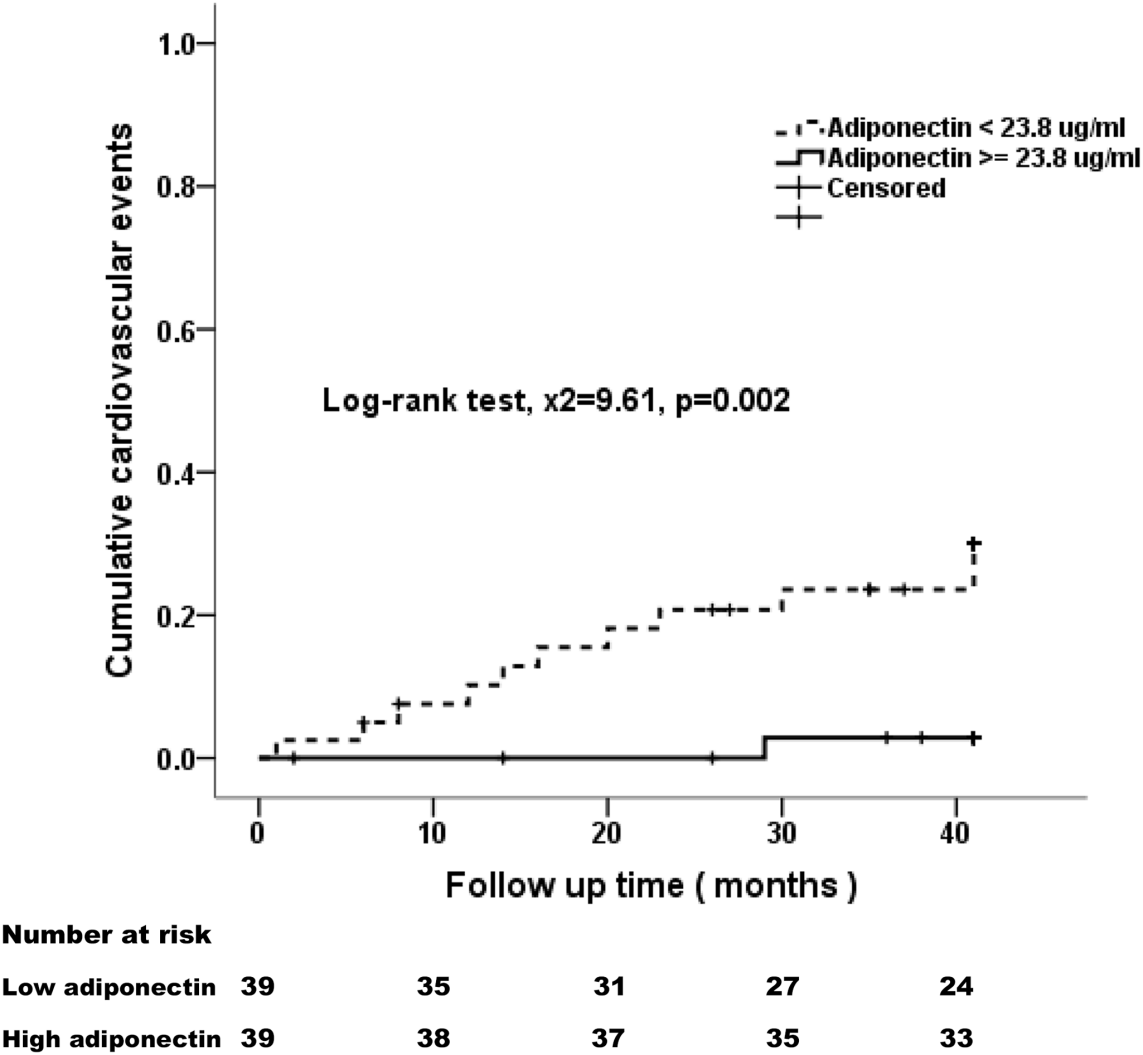
Kaplan-Meier analyses of cumulative probability of cardiovascular events among PD patients during the 3.5-year follow-up. Patients were divided into low and high adiponectin groups according to the median value (23.8 μg/ml).

**Fig 4:**
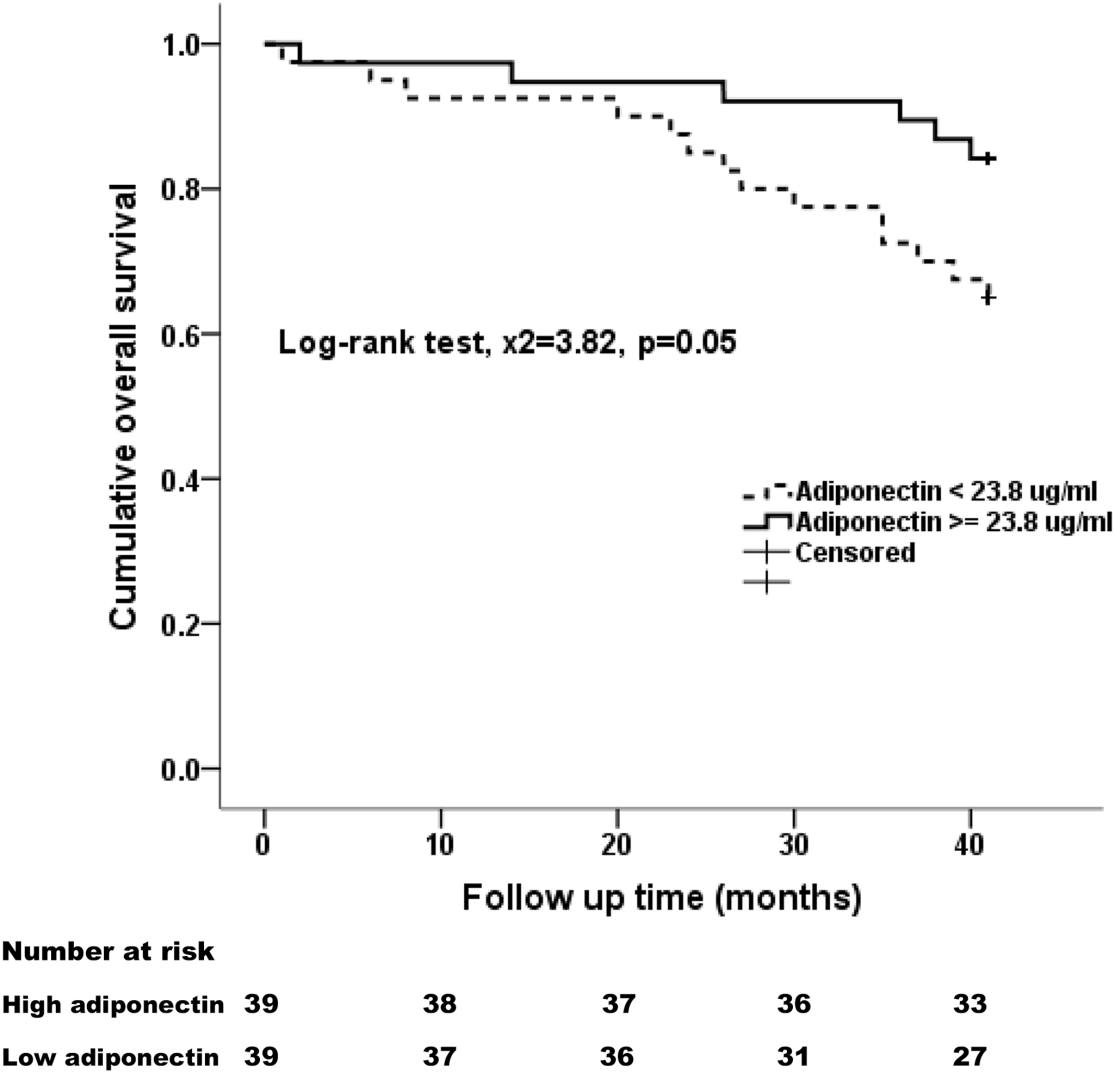
Kaplan-Meier survival curve based on plasma ADPN levels among PD patients in the 3.5-year follow-up. Patients were divided into low and high adiponectin groups according to the median value (23.8 μg/ml).

Kaplan-Meier plots were generated by stratifying the plasma ADPN concentration at the median value (23.8 μg/ml). Patients with higher ADPN values had less CV events (Log-rank test, x^2^ = 9.61, p = 0.002) (Fig 3) and better survival (Log-rank test, x^2^ = 3.82, p = 0.05) (Fig 4). Cox’s proportional hazard model was further applied to assess the independent predictors of CV events and all-cause mortality in our cohort. Factors affecting the development of CV events are listed in Table 4. Lower ADPN, higher hs-CRP levels, older age, history of CV diseases, lower diastolic blood pressure, lower serum albumin, BUN, creatinine, and lower phosphorus levels were associated with cardiac events during the 42-month follow-up. On multivariate Cox regression analysis, only ADPN level (hazard ratio (HR): 0.93, 95% CI: 0.88–0.98, p = 0.02), age (HR: 1.06, 95% CI: 1.01–1.12, p = 0.01) and history of CV diseases (HR: 4.06, 95% CI: 1.27–13.01, p = 0.02) were identified as independent risk factors for CV events during follow-up. Every 1 μg/mL increase of ADPN level was independently predictive of a 7% reduction of CV events.

**Table 4:** Cox Proportional Hazard Analyses of cardiovascular risk.

**Univariate analysis of cardiovascular risk**		
	HRs (95% CI)	p
Adiponectin, μg/mL	0.94 (0.89–0.98)	0.02
Hs-CRP, mg/L	1.10 (1.00–1.20)	0.05
Age (per year)	1.08 (1.03–1.14)	0.002
Male vs. Female	1.60 (0.51–5.05)	0.42
Duration of PD (per month)	0.99 (0.98–1.01)	0.57
History of DM	2.53 (0.82–7.85)	0.11
History of HTN	1.05 (0.29–3.89)	0.94
History of CVD	4.49 (1.12–18.08)	0.04
Body mass index, kg/m^2^	1.01 (0.86–1.19)	0.93
Systolic blood pressure, mmHg	0.98 (0.95–1.02)	0.37
Diastolic blood pressure, mmHg	0.95 (0.90–1.00)	0.05
D4/D0 Glucose	0.56 (0.00–23092.18)	0.91
Peritoneal Kt/V	1.04 (0.22–4.97)	0.96
Renal Kt/V	0.77 (0.14–4.14)	0.76
Total Kt/V	0.72 (0.10–5.28)	0.74
nPNA (gm/Kg/day)	0.17 (0.01–2.37)	0.19
White blood cell count, 10^3^ cells/uL	0.96 (0.79–1.16)	0.66
Hemoglobin, g/dL	1.14 (0.77–1.69)	0.50
Albumin, g/dL	0.29 (0.09–0.94)	0.04
Total cholesterol, mg/dL	1.01 (0.99–1.02)	0.29
LDL-C, mg/dL	1.01 (1.00–1.03)	0.12
HDL-C, mg/dL	0.97 (0.93–1.01)	0.11
Triglyceride, mg/dL	1.00 (0.99–1.01)	0.34
Hemoglobin A1c, %	1.38 (0.97–1.96)	0.07
Fasting glucose, mg/dL	1.01 (1.00–1.02)	0.12
BUN, mg/dL	0.96 (0.93–1.00)	0.04
Creatinine, mg/dL	0.79 (0.65–0.97)	0.03
Uric acid, mg/dL	1.11 (0.74–1.65)	0.62
Calcium, mg/dL	0.99 (0.50–1.98)	0.98
Phosphorus, mg/dL	0.52 (0.28–0.96)	0.04
Ferritin, μg/L	1.00 (1.00–1.01)	0.54
i-PTH, pg/mL	1.00 (1.00–1.01)	0.73
Calcium channel blocker	1.25 (0.17–4.87)	0.75
ARB/ACEIs	0.83 (0.32–2.50)	0.79
Beta-blocker	1.05 (0.28–3.92)	0.95
Anti-platelet agents	0.86 (0.32–3.23)	0.48
Statins	1.59 (0.45–5.64)	0.47
Calcitriol	0.41 (0.10–1.58)	0.19
EPO, Units/month	1.00 (1.00–1.00)	0.86
**Multivariate analysis of cardiovasular risk**		
	HRs (95% CI)	p
Adiponectin, μg/mL	0.93 (0.88–0.98)	0.02
Age (per year)	1.06 (1.01–1.12)	0.01
History of CVD	4.06 (1.27–13.01)	0.02

Hs-CRP, high sensitivity C-reactive protein at baseline; PD, peritoneal dialysis; DM, diabetes mellitus; HTN, hypertension; CVD, cardiovascular disease; nPNA, normalized protein nitrogen appearance; LDL-C, low-density lipoprotein; HDL-C, high-density lipoprotein; BUN, blood urea nitrogen; i-PTH, intact-parathyroid hormone; ARB/ACEI, angiotensin receptor blockade/angiotensin converting enzyme inhibitors; EPO, erythropoietin.

The results of univariate survival analysis demonstrated that all-cause mortality was related to lower ADPN level, higher hs-CRP, older age, history of CV diseases, lower diastolic blood pressure, higher peritoneal Kt/V, lower Renal Kt/V, lower albumin and phosphorus levels (Table 5). Multivariate analysis was then performed in a backward stepwise manner, including parameters with p< 0.10 in univariate analysis, disclosed hs-CRP (HR: 1.11, 95% CI:1.001–1.22, p = 0.04), age (HR: 1.08, 95% CI: 1.02–1.13, p = 0.007), existence of residual renal function (renal Kt/V) (HR: 0.63, 95% CI: 0.41–0.95, p = 0.03) and albumin (HR: 0.29, 95% CI: 0.11–0.79, p = 0.02) were significant predictors of morality (Table 5). Every 1 mg/L increase of hs-CRP level was associated with a 11% increase of all-cause mortality.

**Table 5:** Cox Proportional Hazard Analyses of all-cause mortality.

**Univariate analysis for all cause mortality**		
	HRs (95% CI)	p
Adiponectin, μg/mL	0.96 (0.94–1.00)	0.05
hs-CRP, mg/L	1.09 (1.01–1.17)	0.03
Age (per year)	1.08 (1.04–1.12)	<0.001
Male vs. Female	2.04 (0.82–5.12)	0.13
Duration of PD (per month)	1.01 (0.999–1.02)	0.08
History of DM	1.62 (0.66–3.97)	0.29
History of HTN	1.07 (0.39–2.95)	0.89
History of CVD	3.39 (1.35–8.52)	0.01
Body mass index, kg/m^2^	1.06 (0.94–1.19)	0.38
Systolic blood pressure, mmHg	0.98 (0.95–1.01)	0.16
Diastolic blood pressure, mmHg	0.95 (0.91–0.98)	0.01
D4/D0 Glucose	0.05 (0.00–280.13)	0.50
Peritoneal Kt/V	3.70 (1.05–12.99)	0.04
Renal Kt/V	0.08 (0.00–0.40)	0.02
Total Kt/V	0.69 (0.15–3.29)	0.65
nPNA (gm/Kg/day)	0.39 (0.06–2.83)	0.36
White blood cell count, 10^3^ cells/uL	1.03 (0.99–1.13)	0.12
Hemoglobin, g/dL	0.96 (0.70–1.32)	0.82
Albumin, g/dL	0.31 (0.12–0.78)	0.01
Total cholesterol, mg/dL	0.99 (0.98–1.01)	0.56
LDL-C, mg/dL	1.00 (0.99–1.02)	0.82
HDL-C, mg/dL	0.98 (0.95–1.01)	0.22
Triglyceride, mg/dL	1.00 (0.99–1.01)	0.34
Hemoglobin A1c, %	1.15 (0.83–1.60)	0.39
Fasting glucose, mg/dL	1.01 (0.99–1.01)	0.19
BUN, mg/dL	0.98 (0.95–1.01)	0.12
Creatinine, mg/dL	0.89 (0.77–1.03)	0.11
Uric acid, mg/dL	0.93 (0.67–1.29)	0.64
Calcium, mg/dL	1.17 (0.69–1.98)	0.57
Phosphorus, mg/dL	0.56 (0.35–0.89)	0.02
Ferritin, μg/L	1.00 (1.00–1.01)	0.10
i-PTH, pg/mL	1.00 (0.99–1.00)	0.61
Calcium channel blocker	1.14 (0.38–3.45)	0.81
ARB/ACEIs	1.63 (0.60–4.41)	0.34
Beta-blocker	0.93 (0.33–2.64)	0.89
Anti-platelet agents	2.02 (0.47–8.72)	0.35
Statins	1.70 (0.65–4.45)	0.28
Calcitriol	0.67 (0.25–1.76)	0.28
EPO, Units/month	1.00 (1.00–1.00)	0.24
**Multivariate analysis of all cause mortality**		
	HRs (95% CI)	p
Hs-CRP, mg/L	1.11 (1.001–1.22)	0.04
Age (per year)	1.08 (1.02–1.13)	0.007
Renal Kt/V	0.63 (0.41–0.95)	0.03
Albumin, g/dL	0.29 (0.11–0.79)	0.02

Hs-CRP, high sensitivity C-reactive protein at baseline; PD, peritoneal dialysis; DM, diabetes mellitus; HTN, hypertension; CVD, cardiovascular disease; nPNA, normalized protein nitrogen appearance; LDL-C, low-density lipoprotein; HDL-C, high-density lipoprotein; BUN, blood urea nitrogen; i-PTH, intact-parathyroid hormone; ARB/ACEI, angiotensin receptor blockade/angiotensin converting enzyme inhibitors; EPO, erythropoietin.

## Discussion

Adiponectin as an insulin sensitizer can effectively enhance glucose uptake and lipid metabolism. Similar favorable lipid profile patterns observed in prior studies of dialysis patients [16,17], we found that ADPN levels is inversely associated with TG and positively with HDL-C. Same as previous studies [16], the relationship between ADPN and LDL-C was insignificant in our uremic patients. Additionally, high D4/D0 glucose ratios were associated with low plasma ADPN concentrations. Though the ADPN levels positively correlated with faster peritoneal transport, daily removal of ADPN is minimal according to prior studies [17]. Furthermore, the multiple linear regression analysis disclosed that ADPN was inversely correlated with PD duration. In one randomized, multi-center study of 125 incident PD patients by Lai et al. [29], those on biocompatible PD solutions low in glucose degradation products had elevated serum and effluent ADPN levels, suggesting that peritoneal cavity may be the source of adipokines. Rodriguez at al. had postulated bio-incompatible solutions markedly disturbed secretion patterns of these appetite-regulatory hormones, such as elevation of leptin, reduction of ADPN and acylated ghrelin [30]. Acidic milieu has also been shown to lower circulating ADPN levels through inhibition of ADPN gene transcription in adipocytes [31]. Furthermore, one PD animal study demonstrated oxidative stress induced by glucose-based solutions may suppress ADPN synthesis through pathological changes in abdominal fat tissue [32]. In accordance with aforementioned studies and our findings, longer exposure to conventional glucose- or lactate-based solutions might reduce ADPN levels.

According to the large population-based study from the Framingham database, increment in BMI exponentially increases the risk of dyslipidemia, insulin resistance and subsequently the risk of coronary heart disease, heart failure and ischemic stroke [33]. However, the effect of overweight or obesity has been repeatedly associated with improved survival in HD patients [34,35] but less clear in PD patients with mixed results [36,37]. With these paradoxically epidemiological data, it should be noted that BMI is not a very precise body composition or nutritional parameters. In our study, BMI is an independently negative determinant of plasma ADPN level. However, there was no relations about BMI and CV events and survival outcomes by simple and multivariate Cox regression analysis. Indeed, some authors have proposed low BMI among high ADPN population was linked with unfavorable body anthropometric characteristics, such as loss of lean body mass and body fat in patients on maintenance dialysis [20,23]. Previous studies had disclosed ADPN levels in plasma are negatively regulated by accumulation of visceral, not total body fat [38]. Further studies would be needed to clarify the differential effects of ADPN on body compositions in this special population.

A negative and independent association between hs-CRP and ADPN levels was clearly elucidated in our PD population. Elevation of serum CRP has been found to be a good predictor of various CV outcomes, such as myocardial infarction and stroke in the general population [39] and in individuals undergoing dialysis [27,40]. A reciprocal association of ADPN and CRP levels was observed in type 2 diabetic patients [41] and general population [25,42]. Similar association was shown in HD [43] and PD patients [17,22,44] by cross-sectional analysis. Our study results also indicated that gender is a potent modifier in the interaction between hs-CRP and ADPN. Though no difference was found about the expression levels of serially followed hs-CRP, there were stronger and more negative correlations between ADPN and hs-CRP. The difference of adiposity, insulin resistance and sex hormones may play an important role in this finding [45]. We further analyzed the relations between ADPN levels and longitudinal hs-CRP changes. There was still good association between baseline ADPN and hs-CRP at one- and 2-year follow-up. PD patients with higher ADPN concentrations could maintain significantly lower hs-CRP levels in the first 2 years. The finding of less significantly lower hs-CRP at 3-year follow-up may be partly explained by reduced study numbers. Recent studies have suggested that ADPN may exert anti-inflammatory effect through suppressing the expression of adhesion molecules [46], and inhibiting endothelial NF-κB signaling pathway [47]. Increased dialysate-to-serum ratios of inflammatory mediators, such as tumor necrosis factor-α, interleukin-6, interleukin-8 and CRP, after exposure to less biocompatible dialysate was associated with reduction of ADPN levels [29]. Additionally, it was noticed that inflammatory mediators inhibit ADPN gene expression in cultured adipocytes [48]. Thus, local inflammation within peritoneal cavity and systemic inflammation in then uremic milieu may be causes of lowering plasma ADPN in patients on PD. Aforementioned results warrant further well-designed studies to investigate the mutual interference between ADPN and chronic inflammation in PD patients.

Furthermore, our prospective study confirmed that hyperadiponectinemia is a predictor of better CV outcomes in PD population, though less significant in predicting all-cause mortality than hs-CRP. Despite the inverse relationship between dialysis duration and ADPN concentrations, there was no difference between high and low ADPN groups. Body compositions possibly modify the protective effect of ADPN on all-cause mortality in dialysis patients. Association of ADPN and decreased hazard of death was seen only in participants with BMI≧24 kg/m^2^ [49]. Accordingly, the findings of reverse association of BMI and ADPN and lower BMI among high ADPN group may partly explain reduced impact of hyperadiponectinemia on survival outcome in our study cohort. Rhee at al. [20] recently reported apposite results with association between higher ADPN concentration and decreased survival among 501 HD patients after further adjustment of anthropometric confounders. Lack of CV disease history and older dialysis vintage in the highest ADPN tertile may explain this discrepancy of CV and death outcomes. In our study, history of CV disease is a significant risk factor for cardiovascular events. Since ADPN exhibits anti-atherogenic properties, therapies aimed at raising ADPN levels could be potentially beneficial in the prevention or treatment of CV diseases in patients undergoing PD. In a randomized crossover trial [50], oral pioglitazone 15 mg once daily significantly improved insulin resistance, reduced inflammation and increased ADPN in PD patients. Use of Extraneal (7.5% icodextrin) solution overnight [51] or biocompatible PD fluid regimens [52], (consisted of 1 exchange of Physioneal (1.5% glucose, PH:7.4), 1 exchange of Nutrineal (1.1% amino acid), and 1 overnight exchange of Extraneal) led to increase of systemic ADPN levels.

There are several limitations of our study. First, we did not use body composition analysis techniques (such as bioelectric impedance, dual-emission X-ray absorptiometry of the whole body and computed tomography of the abdomen, etc) to clarify the impact of adiposity on the survival and metabolic dys-regulation in our study population. Second, we just performed single-point measurements of ADPN in prevalent PD patients, thus there was no really baseline data and changes of ADPN over time was not considered. Third, due to the small number of patients who died, specific CV disease related mortality risk was not analyzed. Fourth, our analyses did not take into consideration components of PD solutions. Finally, we did not examine residual confounders, such as insulin resistance and other inflammatory markers, etc.

## Conclusions

This prospective cohort study demonstrated the importance of ADPN and hs-CRP in predicting CV events and all-cause mortality in PD population during 3.5-year follow-up period after comprehensive adjustment of possible confounders. Plasma ADPN was positively correlated with HDL-C and negatively associated with hs-CRP, BMI, fast peritoneal transport, TG and duration of PD therapy independently. We believe it was the first study to show the strong relations of ADPN and longitudinal hs-CRP changes in patients undergoing PD. Serum hs-CRP levels were noticed consistently lower in the high ADPN group during 2-year follow-up.

## Supporting Information

S1 Table Clinical and biochemical characteristics of participants based on gender. (DOC)

S2 Table Relationship between adiponectin, baseline characteristics and annual hs-CRP data according to gender. (DOC)
