# Supplementary material for: Association of Adiponectin with High-Sensitivity C-Reactive Protein and Clinical Outcomes in Peritoneal Dialysis Patients: A 3.5-Year Follow-Up Study
Source: PLoS One. 2015 Oct 16;10(10):e0141058. doi: 10.1371/journal.pone.0141058 (PMC4608701; doi:10.1371/journal.pone.0141058)
Supplement: S1 Table — (DOC) [file pone.0141058.s001.doc]

**S1 Table. Clinical and biochemical characteristics of participants based on gender**

|  | | **Male (N=39)** | **Female (N=39)** | p value |
| --- | --- | --- | --- | --- |
| Adiponectin, μg/mL | | 26.99±17.17 | 31.93±18.71 | 0.16 |
| **Demographics** | | | | |
| Age, years | | 50.95±14.06 | 53.33±11.74 | 0.48 |
| Duration of PD, months | | 35.44±21.50 | 43.56±36.34 | 0.11 |
| Body mass index, kg/m^2^ | | 23.88±3.50 | 22.14±3.01 | 0.06 |
| Systolic blood pressure, mmHg | | 133.46±18.67 | 135.92±17.98 | 0.70 |
| Diastolic blood pressure, mmHg | | 80.33±9.58 | 80.15±10.92 | 0.94 |
| **Concomitant diseases, % (n)** | | | | |
| Diabetes mellitus | | 35.9 (14) | 25.6 (10) | 0.33 |
| Hypertension | | 74.4 (29) | 74.4 (29) | 1.00 |
| Ischemic heart disease/stroke | | 17.9 (7) | 12.8 (5) | 0.53 |
| **Dialysis Parameters** | | | | |
| *Adequacy of dialysis* |  | | | |
| Solute clearance (Total Kt/V) | | 1.99±0.21 | 2.24±0.34 | <0.01 |
| Peritoneal Kt/V | | 1.78±0.30 | 1.93±0.42 | 0.33 |
| Residual GFR (ml/min per 1.73 m^2^) | | 1.75±2.43 | 1.70±2.40 | 0.68 |
| Renal Kt/V | | 0.21±0.26 | 0.31±0.48 | 0.90 |
| nPNA (gm/Kg/day) | | 1.01±0.19 | 1.17±0.25 | <0.01 |
| Daily Urine Amount, mL | | 336.22±341.58 | 357.95±422.29 | 0.79 |
| *Peritoneal solute transport rate* |  | | | |
| D4/D0 Glu | | 0.39±0.55 | 0.39±0.56 | 0.99 |
| 4-h D/P Cr | | 0.67±0.09 | 0.66±0.09 | 0.73 |
| **Biochemical parameters** | | | | |
| White blood cell count, 10^3^ cells/uL | | 7.62±1.69 | 8.85±5.45 | 0.77 |
| Hemoglobin, g/dL | | 10.34±1.67 | 9.73±1.18 | 0.09 |
| Albumin, g/dL | | 3.88±0.45 | 3.75±0.42 | 0.37 |
| Total cholesterol, mg/dL | | 174.38±35.34 | 194.36±45.21 | 0.04 |
| LDL-C, mg/dL | | 97.97±32.43 | 112.41±40.38 | 0.09 |
| HDL-C, mg/dL | | 37.65±15.72 | 49.05±15.51 | <0.01 |
| Triglyceride, mg/dL | | 206.38±157.55 | 172.41±96.38 | 0.39 |
| Hemoglobin A1c, % | | 6.06±1.11 | 5.93±1.35 | 0.31 |
| Fasting glucose, mg/dL | | 127.74±50.39 | 122.59±49.40 | 0.36 |
| BUN, mg/dL | | 62.46±15.49 | 61.82±19.13 | 0.56 |
| Creatinine, mg/dL | | 13.89±3.19 | 11.16±2.72 | <0.01 |
| Uric acid, mg/dL | | 7.16±1.53 | 7.04±1.31 | 0.79 |
| Calcium, mg/dL | | 9.28±0.81 | 9.53±0.83 | 0.27 |
| Phosphorus, mg/dL | | 4.83±0.94 | 4.82±1.09 | 0.90 |
| Ferritin, μg/L | | 496.21±316.59 | 648.97±561.33 | 0.33 |
| i-PTH, pg/mL | | 249.39±250.27 | 300.38±260.70 | 0.35 |
| **Medications, % (n)** | | | | |
| Calcium channel blocker | | 59.0 (23) | 46.2 (18) | 0.26 |
| ARB/ACEI | | 46.2 (18) | 35.9 (14) | 0.36 |
| Beta-blocker | | 53.8 (21) | 46.2 (18) | 0.49 |
| Statins | | 38.5 (15) | 48.7 (19) | 0.36 |
| Anti-platelet agents | | 23.1 (9) | 10.3 (4) | 0.13 |
| Calcitriol | | 35.9 (14) | 43.6 (17) | 0.49 |
| Hs-CRP_0 | | 4.56±5.14 | 3.60±3.56 | 0.60 |
| Hs-CRP_1 | | 3.83±3.17 | 4.49±5.59 | 0.99 |
| Hs-CRP_2 | | 4.34±5.01 | 4.39±4.85 | 0.96 |
| Hs-CRP_3 | | 3.89±2.97 | 4.68±4.53 | 0.76 |

Values expressed as mean ± standard deviation or percentage (number)

Abbreviations: PD, peritoneal dialysis; GFR, glomerular filtration rate; nPNA, normalized protein nitrogen appearance; 4-h D/P Cr, dialysate/plasma creatinine ratio at 4 hours; LDL-C, low-density lipoprotein; HDL-C, high-density lipoprotein; BUN, blood urea nitrogen; i-PTH, intact-parathyroid hormone; ARB/ACEI, angiotensin receptor blockade/angiotensin converting enzyme inhibitors; hs-CRP_0, high sensitivity C-reactive protein at baseline; hs-CRP_1, high sensitivity C-reactive protein at one-year follow up; hs-CRP_2, high sensitivity C-reactive protein at 2-year follow up; hs-CRP_3, high sensitivity C-reactive protein at 3-year follow up
