# Supplementary material for: Association of Adiponectin with High-Sensitivity C-Reactive Protein and Clinical Outcomes in Peritoneal Dialysis Patients: A 3.5-Year Follow-Up Study
Source: PLoS One. 2015 Oct 16;10(10):e0141058. doi: 10.1371/journal.pone.0141058 (PMC4608701; doi:10.1371/journal.pone.0141058)
Supplement: S2 Table — (DOC) [file pone.0141058.s002.doc]

**S2 Table. Relationship between adiponectin, baseline characteristics and annual hs-CRP data according to gender**

|  | **Male (N=39)** |  | **Female (N=39)** | |  | |
| --- | --- | --- | --- | --- | --- | --- |
|  | **Pearson r*** | **p value** | **Pearson r*** | | **p value** | |
| **Demographics** | | | | | | |
| Age, years | -0.414 | <0.01 | | -0.534 | | <0.01 |
| Duration of PD, months | -0.224 | 0.09 | | -0.294 | | 0.04 |
| Body mass index, kg/m^2^ | -0.390 | <0.01 | | -0.368 | | 0.01 |
| Systolic blood pressure, mmHg | 0.336 | 0.02 | | 0.066 | | 0.35 |
| Diastolic blood pressure, mmHg | 0.257 | 0.06 | | 0.523 | | <0.01 |
| **Dialysis Parameters** | | | | | | |
| Solute clearance (Total Kt/V) | -0.140 | 0.21 | | -0.306 | | 0.03 |
| Peritoneal Kt/V | -0.227 | 0.09 | | 0.009 | | 0.48 |
| Residual GFR (ml/min per 1.73 m^2^) | 0.066 | 0.35 | | -0.214 | | 0.10 |
| Renal Kt/V | 0.144 | 0.19 | | -0.224 | | 0.09 |
| nPNA (gm/Kg/day) | 0.142 | 0.20 | | 0.137 | | 0.20 |
| Daily Urine Amount, mL | 0.413 | <0.01 | | 0.072 | | 0.33 |
| D4/D0 Glu | -0.316 | <0.01 | | -0.136 | | 0.21 |
| 4-h D/P Cr | 0.471 | 0.03 | | -0.070 | | 0.34 |
| **Biochemical parameters** | | | | | | |
| White blood cell count, 10^3^ cells/uL | -0.123 | 0.23 | | -0.062 | | 0.35 |
| Hemoglobin, g/dL | 0.051 | 0.38 | | 0.075 | | 0.33 |
| Albumin, g/dL | -0.230 | 0.09 | | -0.008 | | 0.48 |
| Total cholesterol, mg/dL | 0.070 | 0.34 | | -0.030 | | 0.43 |
| LDL-C, mg/dL | 0.148 | 0.19 | | 0.053 | | 0.38 |
| HDL-C, mg/dL | 0.337 | 0.02 | | 0.337 | | 0.02 |
| Triglyceride, mg/dL | -0.247 | 0.07 | | -0.502 | | <0.01 |
| Hemoglobin A1c, % | 0.137 | 0.21 | | -0.180 | | 0.14 |
| Fasting glucose, mg/dL | 0.062 | 0.36 | | -0.131 | | 0.21 |
| BUN, mg/dL | -0.075 | 0.33 | | 0.316 | | 0.03 |
| Creatinine, mg/dL | -0.113 | 0.25 | | 0.175 | | 0.14 |
| Uric acid, mg/dL | -0.345 | 0.02 | | -0.148 | | 0.19 |
| Calcium, mg/dL | -0.363 | 0.01 | | -0.224 | | 0.09 |
| Phosphorus, mg/dL | -0.179 | 0.14 | | 0.209 | | 0.10 |
| Ferritin, μg/L | -0.158 | 0.17 | | -0.330 | | 0.02 |
| i-PTH, pg/mL | -0.142 | 0.19 | | -0.016 | | 0.46 |
| ln hs-CRP_0 | -0.342 | 0.02 | | -0.477 | | <0.01 |
| ln hs-CRP_1 | -0.262 | 0.05 | | -0.531 | | <0.01 |
| ln hs-CRP_2 | -0.291 | 0.04 | | -0.388 | | <0.01 |
| ln hs-CRP_3 | -0.164 | 0.22 | | -0.220 | | 0.13 |

* r was calculated by Pearson correlation analysis

Abbreviations: PD, peritoneal dialysis; GFR, glomerular filtration rate; nPNA, normalized protein nitrogen appearance; 4-h D/P Cr, dialysate/plasma creatinine ratio at 4 hours; LDL-C, low-density lipoprotein; HDL-C, high-density lipoprotein; BUN, blood urea nitrogen; i-PTH, intact-parathyroid hormone; ARB/ACEI, angiotensin receptor blockade/angiotensin converting enzyme inhibitors; hs-CRP_0, high sensitivity C-reactive protein at baseline; hs-CRP_1, high sensitivity C-reactive protein at one-year follow up; hs-CRP_2, high sensitivity C-reactive protein at 2-year follow up; hs-CRP_3, high sensitivity C-reactive protein at 3-year follow up
